# Application of Sugar Cane Bagasse Hydrochar for Fipronil and Atrazine Removal from Water

**DOI:** 10.1021/acsomega.5c06438

**Published:** 2025-11-12

**Authors:** Avenancia Tavares Belo de Carvalho, Luan de Souza Leite, Beatriz De Caroli Vizioli, Sandro José de Andrade, Márcia Cristina Bisinoti, Cassiana Carolina Montagner

**Affiliations:** † Environmental Chemistry Laboratory, Institute of Chemistry, 28132 University of Campinas, Campinas, São Paulo 13083-970, Brazil; ‡ Institute of Physics and Chemistry, Federal University of Itajubá, Itajubá, Minas Gerais 37500-903, Brazil; § Institute of Biosciences, Humanities and Exact Sciences, São Paulo State University (UNESP), São José Do Rio Preto, São Paulo 15054-000, Brazil

## Abstract

Pesticides are persistent environmental pollutants that pose risks to human health and ecosystems while also contributing to the contamination of waterways. Conventional water treatment technologies are often ineffective in removing pesticides. Therefore, several treatments have been developed to prevent these compounds from being present in the finished water. In this context, a hydrochar synthesized via hydrothermal carbonization of sugar cane bagasse and vinasse was evaluated to remove atrazine (ATZ) and fipronil (FIP) from water. Kinetic data for ATZ and FIP were successfully fitted using the Elovich (*R* ^2^ = 0.937) and PFO models (*R* ^2^ = 0.791), respectively. The adsorption equilibrium was reached at 48 h for ATZ and 1 h for FIP. The Langmuir, Freundlich, and Sips isotherms accurately described the experimental data for both pesticides (*R* ^2^ > 0.93), with maximum adsorption capacities of 273.3 μg g^–1^ for ATZ and 34.6 μg g^–1^ for FIP. The adsorption decreased with increasing pH, while ATZ adsorption was only slightly affected. The adsorption mechanism was mainly driven by hydrophobic interaction, hydrogen bonding, and π–π interactions. This study demonstrates the potential of sugar cane-derived hydrochar as a low-cost adsorbent for removing ATZ and FIP from water. Further studies are recommended to improve the hydrochar’s affinity for these pesticides.

## Graphical abstract

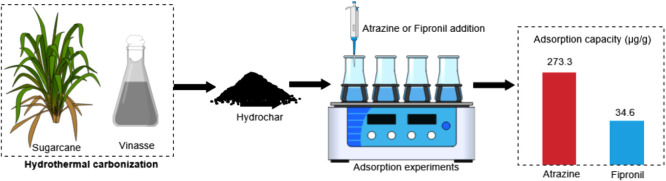

## 1 Introduction

Pesticides are essential in modern agriculture, safeguarding crops from pests, diseases, and weeds that threaten yield and quality. These compounds help meet global food demand by reducing the risk of crop failures.^1^ Among pesticide classes, insecticides and herbicides are widely applied in agricultural activities, including in Brazil, the world’s largest consumer of pesticides. However, their indiscriminate use leads to environmental challenges, including the pollution of soil and water resources, as well as potential health risks to humans and wildlife.^2,3^

It has been estimated that up to 10% of the total pesticide concentration applied to the field can reach the water bodies by transport and degradation processes.^4^ Consequently, these compounds are often detected in water matrices. For example, Acayaba et al. (2021)^5^ detected a significant amount of pesticides in surface and groundwater samples from the region with the world’s largest sugar cane production. Atrazine (ATZ) is the most frequently detected herbicide in water sources, with concentrations ranging from 0.001 to 2.74 μg L^–1^.^5–7^ Fipronil (FIP), one of the most popular insecticides, is detected at concentrations varying from 0.01 to 26.20 μg L^–1^.^8,9^ Both chemicals have raised concerns despite their effectiveness in agricultural activities. FIP is classified as WHO Class II (moderately hazardous), while ATZ is classified as WHO Class III (slightly hazardous).^3^ Therefore, these compounds and their byproducts are included in drinking water standards worldwide to prevent harm to human health. As an example, the Brazilian drinking water standard (Regulation 888/2021) limits the ATZ and its byproducts (deethylatrazine, deisopropylatrazine, and deethyl-deisopropylatrazine) concentration to 2.0 μg L^–1^ and FIP concentration to 1.2 μg L^–1^.^10^

Conventional technologies applied to water treatment, including chemical coagulation, flocculation, sedimentation or flotation, and rapid filtration, are ineffective in removing pesticides. Vizioli et al. (2023)^6^ monitored the concentration of ATZ and three byproducts in two drinking water treatment plants in Brazil, and no significant removal was found after the treatment. Thus, alternative water treatment technologies are necessary to effectively remove such compounds and minimize their occurrence in drinking water. Different technologies have shown efficacy in removing pesticides from water in the literature, including advanced oxidation processes,^11^ adsorption,^12–14^ and membrane filtration.^15^

Adsorption is an effective method for removing pesticides from water due to its simplicity and high efficiency. However, activated carbon, a popular adsorbent, is costly, accounting for up to 70% of the total chemical cost, making it impractical for water treatment, especially in small facilities.^16^ In this context, the studies have focused on synthesizing low-cost adsorbents derived from sustainable materials or industrial waste, aligning with principles of green chemistry by promoting eco-friendly, efficient, and economical solutions for pollution control. Several adsorbents have been tested to remove FIP or ATZ from water, such as araçá fruit husks,^17^ açaí pulp,^12^ banana peel,^14^ corn straw,^18^ hackberry seeds,^13^ rice husk,^19,20^ sugar cane bagasse ash,^16^ and wood industry byproducts.^21^

Sugar cane bagasse and vinasse are wastes primarily generated by the Brazilian sugar cane industry. Bagasse has emerged as a versatile and sustainable resource for developing advanced materials in catalysis and chemical synthesis. Its transformation into carbon-rich and functionalized materials enables its application as an adsorbent, catalyst, catalyst support, and anchoring medium for active species, owing to its high surface area, porosity, and abundance of functional groups.^22–24^ Additionally, these materials facilitate the conversion of biomass into valuable platform chemicals, such as levulinic and formic acid, supporting sustainable biorefinery processes and contributing to waste valorization and green chemistry initiatives.^25^

In this context, the reuse of sugar cane bagasse and vinasse in producing adsorbents for removing water pollutants appears promising and has been tested for metal removal from water.^22,23^ However, a combination of these two wastes has not been tested for pesticide adsorption, despite having a high specific surface area, porosity, and abundant surface functional groups.^26,27^ It could be attractive considering the Brazilian scenario of intense agriculture with pesticide application and a large volume of waste generation from the sugar cane industry. In this scene, an adsorbent from sugar cane bagasse and vinasse obtained by the hydrothermal carbonization process was applied for ATZ and FIP removal from water. The hydrochar was characterized by FTIR spectroscopy, morphology, and surface analyses. Adsorption tests were conducted to investigate the effect of pH, adsorption kinetics, and isotherms of the process.

## 2 Material And Methods

### 2.1 Materials

Samples of concentrated vinasse and sugar cane bagasse were obtained from a sugar cane industry in São Paulo State, Brazil. Before use, both waste materials underwent pretreatment. The vinasse was homogenized through stirring, while the bagasse was air-dried at room temperature, ground using a knife mill, and sieved (ASTM #35, particle diameter <0.5 mm). The samples were then characterized and published in previous studies. Bagasse was composed of 37.2% C, 5.8% H, 50.8% O, and 6.49% ash, and the dry vinasse of 35.2% C, 6.1% H, 39.3% O, 3.0% N, 0.9% S, and 19.4% ash.^28^

High purity standards of ATZ (99.1%, CAS #1912–24–9) and FIP (98.8%, CAS #120068–37–3) were purchased from Merck (Darmstadt, Germany). Stock solutions were prepared in methanol at concentrations ranging from 300 to 500 mg L^–1^. The working solutions (10 mg L^–1^) were prepared by diluting the stock solution in ultrapure water. Ultrapure water was collected from Millipore’s Synergy Water Purification System (Burlington, USA).

Methanol (99.9%, CAS #67–56–1) and phosphoric acid (85%, CAS #7664–38–2) were purchased from Merck (Darmstadt, Germany), hydrochloric acid (37%, CAS #7647–01–0) was purchased from Mallinckrodt (London, UK), sodium hydroxide (CAS #1310–73–2) was purchased from Allkimia (Campinas, Brazil), and acetonitrile (99.9%, CAS #75–05–8) was purchased from Sigma-Aldrich (Burlington, USA). Hydrophobic PTFE syringe filters (13 mm diameter, 0.22 μm pore size) were purchased from Analytica (São Paulo, Brazil).

### 2.2 Hydrochar Synthesis

The hydrochar was obtained through a hydrothermal carbonization process, as described in a previous studies.^28,29^ Briefly, a mixture of 3.0 g of sugar cane bagasse, 60 mL of vinasse, and phosphoric acid (4% v•v^–1^) was stirred for 15 min and inserted in a homemade reactor of a Teflon cup (capacity of 80 mL). Phosphoric acid was used as an activating agent to increase the hydrochar’s surface area, porosity, and the abundance of acidic and oxygen-containing functional groups (e.g., carboxyl, hydroxyl, phosphate groups).^27,30^

Then, the reactor was placed in a high-pressure autoclave (capacity of 600 mL) at a temperature of 230 °C. After 13 h, the reactor was collected and placed immediately in the ice bath to stop the reaction.

The obtained hydrochar was separated from the liquid by vacuum filtration and washed with deionized water until it reached a constant pH value (3.3–5.0). Then, the adsorbent was dried at 50 °C until it reached a constant weight. Elemental composition was determined using an elemental analyzer EA1108 (Fisons, USA). The final product had a size of 0.425 mm and a composition of 58.2% C, 7.1% H, 14.6% O, 3.5% N, 0.2% S, and 24.4% ash.^28^

### 2.3 Hydrochar Characterization

The synthesized hydrochar was characterized by FTIR spectroscopy, morphology, and surface analyses. FTIR spectra were obtained using an ATR-FTIR spectrometer (Cary 630, Agilent) in the range of 4000–400 cm^–1^ with a resolution of 1.0 cm^–1^ and number of scans of 64. The spectra were baseline-corrected using Spectragryph software v1.2.16.1 (Oberstdorf, Germany) before spectral comparison. Analyses of surface area and pore distribution were determined by nitrogen adsorption–desorption isotherms using the BET method (Nova4200e, Quantachrome). The total pore volume per gram of hydrochar was determined at a saturation pressure of liquid nitrogen (P/P_0_ = 0.302). The hydrochar was previously dried at 25 °C for 24 h under a vacuum.

Morphology was examined using a Quanta FEG 250 field-emission scanning electron microscope (FEI Co., USA) in environmental scanning electron microscope (ESEM) mode at 130 Pa (water vapor pressure), operated at 10 kV with a gaseous secondary electron detector (GSED) and a working distance of 10 mm. Samples were mounted on conductive carbon tape.

### 2.4 Pesticide Quantification

Pesticide analysis was performed on a Shimadzu SCL-10AVP high-performance liquid chromatograph equipped with an SPD-M10AVP diode array detector and a DGU-14A degasser (Kyoto, Japan), and a manual Rheodyne 7725i injector with a 100 μL injection volume (Bensheim, Germany). Separation was carried out using a Zorbax Eclipse XDB 80Å C18 (4.6 × 150 mm, 5 μm) (Agilent Technologies, Wilmington, USA). The mobile phase consisted of ultrapure water (A) and acetonitrile (B) at a flow rate of 1.0 mL min^–1^. Gradient elution as a function of solvent B was set as follows: from 60% to 100% in 6 min, then from 100% to 60% in 1 min, and maintained at 60% for 5 min. The chromatographic run lasted 12 min, which was sufficient to achieve retention times of 3.1 min for ATZ and 5.8 min for FIP. Detection was carried out at 221 nm.

The analytical method was validated in accordance with Brazilian validation guidelines established by regulatory authorities.^31,32^ The figures of merit evaluated were selectivity, instrumental limit of detection (iLD), instrumental limit of quantification (iLQ), linearity, trueness, precision, and robustness. These parameters ensured the reliability of the method for quantifying the target pesticides under the established chromatographic conditions. Further information regarding the method validation is provided in the Supporting Information.

### 2.5 Adsorption Tests

The adsorption experiments used 20 mg (±0.3%) of hydrochar and 5 mL of pesticide solution (100 μg L^–1^ ATZ or FIP). The mixture was added to 10 mL glass tubes with polytetrafluoroethylene caps and placed on a roto torque shaker (Marconi MA161/ROTO) at room temperature (20 °C) with constant agitation (40 rpm). The sample pH was adjusted using 0.1 mol L^–1^ NaOH or HCl solutions. After the contact time, the supernatant was collected and filtered through 0.22 μm PTFE syringe filters for HPLC-DAD quantification.

Several sets of adsorption tests were performed to check the influence of different parameters on ATZ/FIP adsorption by the hydrochar. The pH effect (4, 7, and 10) was evaluated with the initial ATZ/FIP concentration of 100 μg L^–1^ and contact time of 5 h. Kinetics experiments were conducted with an initial pesticide concentration of 100 μg•L^–1^ and different contact times for ATZ (0.5, 1, 3, 5, 24, 48, 72, and 96 h) and for FIP (0.083, 0.25, 0.75, 1, 3, 6, 24, and 48 h). The isotherm experiments were conducted with a contact time of 48 h for ATZ and 1 h for FIP and with different initial ATZ/FIP concentrations (10, 40, 100, 200, 300, and 400 μg L^–1^). All tests were performed in triplicate. Pesticide concentrations were selected based on a worst-case scenario (a concentration 2 orders of magnitude higher than reported in the literature) considering previous studies in real water matrices.^5–9^

Pesticide removal per hydrochar mass at time *t* (*q*_*t*_, μg g^–1^) was determined using eq 1), where *C*_*i*_ and *C*_*t*_ are the initial concentration and pesticide concentration (ATZ or FIP) at time *t* in the solution (μg L^–1^), respectively, *V* is the volume of pesticide solution (0.005 L), and *m* is the hydrochar mass (0.02 g).

1 $$q_{t}=\left(C_{i}-C_{t}\right)\frac{V}{m}$$

The adsorption was also characterized by FTIR analysis, as described in Section 2.3. Hydrochar samples were collected after the adsorption test, filtered through a 0.22 μm membrane, and dried at 50 °C before analysis.

### 2.6 Zeta Potential

Zeta potential (ZP) measurements were done to evaluate the influence of electrostatic interactions on the adsorption process. Solutions of ATZ, FIP, and hydrochar were prepared at different pH values (4, 7, and 10). The ZP value for each condition was measured using Zetasizer Nano ZS equipment (Zen3600, Malvern) at 20 °C.

### 2.7 Data Fitting

Three kinetic models were used in this study to assess the adsorption rate at which the synthesized hydrochar adsorbs pesticides. The fit of the experimental data to the pseudo-first-order (PFO, eq 2), pseudo-second-order (PSO, eq 3), and Elovich models was evaluated.^33^

2 $$q_{t}=q_{e}\left(1-exp^{-K_{1}t}\right)$$

3 $$q_{t}=\frac{{q_{e}}^{2}K_{2}t}{1+q_{e}K_{2}t}$$

4 $$q_{t}=\frac{1}{b}\ln\left(1+abt\right)$$

Here q_e_ is the amount of pestice adsorbed at the equilibrium (μg g^–1^), *K*_1_ (h^–1^), and *K*_2_ (g μg^–1^ h^–1^) are the constants of the PFO and PSO models, respectively; *a* (μg g^–1^ h^–1^) and *b* (g μg^–1^) are the initial adsorption and desorption rate constant of the Elovich model, respectively.

The relationship between pesticide concentration in the solution and pesticide uptake by the hydrochar at equilibrium can be described by isotherms models. The Langmuir eq 5, Freundlich eq 6, and Sips eq 7 isotherm models were used to model the interaction between the ATZ/FIP and hydrochar.^34^

5 $$q_{e}=\frac{a_{m}K_{L}C_{e}}{1+K_{L}C_{e}}$$

6 $$q_{e}=K_{F}{C_{e}}^{1/n}$$

7 $$q_{e}=\frac{K_{s}{C_{e}}^{\beta s}}{1+a_{s}{C_{e}}^{\beta s}}$$

Here *q*_*e*_ (μg g^–1^) and *C*_*e*_ (μg L^–1^) represent the ATZ/FIP uptake and concentration at equilibrium, respectively; a_m_ is the maximum adsorption capacity of the adsorbent (μg g^–1^); *K*_*L*_ is the Langmuir adsorption constant related to the energy of adsorption (L g^–1^); *K*_*F*_ is the Freundlich adsorption capacity constant [(μg g^–1^)­(L μg^–1^)^1/n^], 1/*n* is the adsorption intensity; and β_*S*_ (−), *K*_*S*_ (L g^–1^), and *a*_*S*_ (L μg^–1^) are constants of the Sips model.

Data fitting was performed using the GRG nonlinear method in Excel Solver. The goodness of fit was evaluated by the coefficient of determination (*R* ^2^), chi-square (χ^2^), and root mean squared error (*RMSE*).^35^

### 2.8 Statistical Analysis

Statistical analyses were conducted using GraphPad Prism v.6.01 (San Diego, USA) and OriginPro 2024 v.10.1.5.132 (Northampton, USA). ANOVA and Tukey’s test were employed to compare hydrochar adsorption among various experimental conditions.

## 3 Results and Discussion

### 3.1 Hydrochar Characterization

The BET surface area, pore diameter, and total pore volume values for the synthesized hydrochar are shown in Table 1. The hydrochar had a surface area of 11.92 m^2^ g^–1^, an average pore diameter of 9.66 Å, and a total pore volume of 5.76 × 10^–3^ cm^3^ g^–1^. The total pore volume was obtained for pores smaller than 13.0 Å, indicating that the hydrochar is 100% microporous (i.e., pore size less than 2 nm).

**Table 1:** Hydrochar Characterization.

Parameters	Hydrochar
BET surface area (m^2^ g^–1^)	11.92
Average pore diameter (Å)	9.66
Total pore volume (cm^3^ g^–1^)	5.76 × 10^–3^
Pore size distribution (%) (micro, meso, macroporous)^a^	100–0–0

a Micropores <2 nm, mesopores 2–50 nm, and macropores >50 nm.

The surface area obtained in the present study is higher than that reported in the literature. Malool et al. (2021)^26^ reported a surface area of 5.99 m^2^ g^–1^ for bagasse submitted to hydrothermal carbonization at 180 °C for 11.5 h with water (5:1 w w^–1^) and ZnCl_2_ (3.5:1 w w^–1^). Zhou et al. (2022)^27^ reported an area of 7.84 m^2^ g^–1^ for hydrochar obtained from bagasse and phosphoric acid (1:19 w w^–1^) at 240 °C for 10 h.

The functional groups on the hydrochar surface were determined by ATR-FTIR analysis (Figure 1). Hydrochar was rich in oxygen-containing functional groups. A broad band around 3441 cm^–1^ is attributed to O–H stretching vibrations associated with carboxyl (−COOH) or hydroxyl (−OH) groups. It is linked to the main components of sugar cane bagasse (hemicellulose, cellulose, and lignin), which were not entirely degraded during hydrothermal carbonization.^27^ The peaks observed in the range of 3000–2800 cm^–1^ were assigned to the aliphatic axial deformation (2919 cm^–1^) and the methoxyl group vibration (2850 cm^–1^) of C–H.^36^ The C = O bonds in the carboxyl and aldehyde groups were observed at 1697 cm^–1^. The stretching vibration peaks corresponding to C = C and C–O bonds in aromatic rings were identified at 1606 and 1513 cm^–1^, respectively. The large and intense band between 1250 and 950 cm^–1^ is attributed to the presence of phosphoric acid during the synthesis process.^28^ Symmetric and asymmetric stretching vibrations of the ether linkage (C–O–C) were observed at 1110 cm^–1^. The peak at 918 cm^–1^ was attributed to the out-of-plane aromatic C–H bending vibrations. These functional groups have been previously reported for sugar cane bagasse under different treatments, such as hydrothermal carbonization^26–28^ and treatment with carbon dioxide.^36^

**Figure 1:**
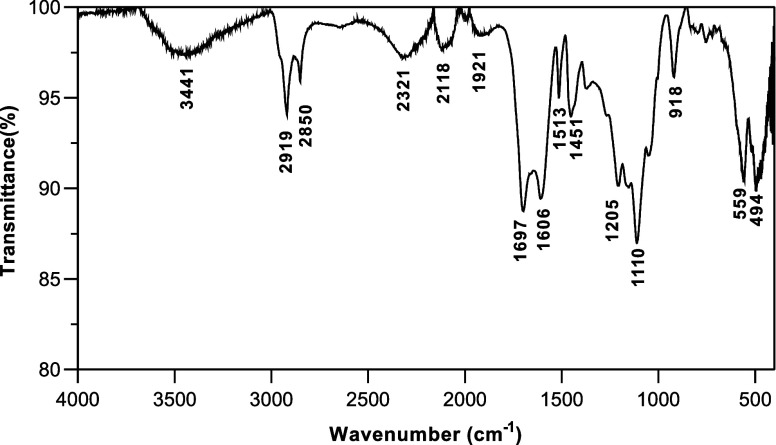
ATR-FTIR spectra of the hydrochar.

The surface morphology was evaluated by SEM analysis (Figure 2). The particles exhibited rigid, spongy, and irregular shapes (Figure 2a), as typically observed in sugar cane bagasse samples.^36^ Hydrothermal carbonization creates spongy structures with irregular cracks and canals on sugar cane bagasse.^26^ It is possible to observe uneven and highly rough areas at 2000× magnification (Figure 2b). However, the presence of cavities and pores is not evident on the hydrochar surface, which may explain the low surface area (11.92 m^2^ g^–1^) quantified by the BET method.

**Figure 2:**
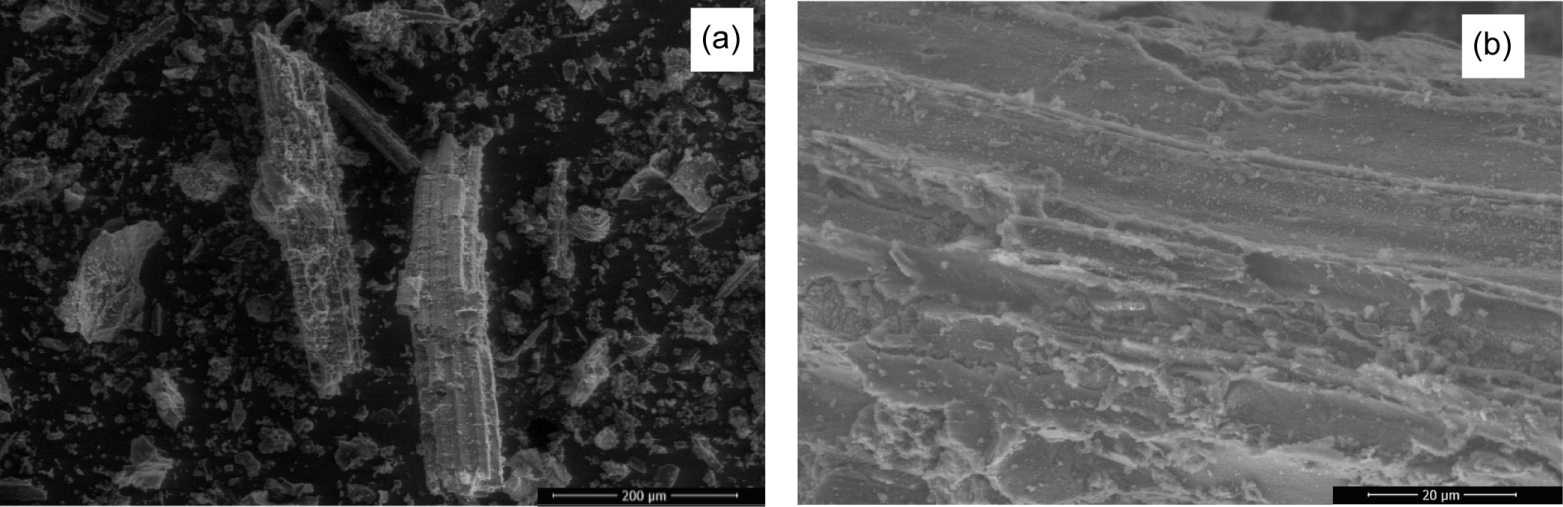
Scanning electron microscopy (SEM) of the hydrochar at (a) 250× and (b) 2000× magnification.

### 3.2 pH Effect

The pH of the pesticide solution affected the adsorption of the two pesticides differently (Figure 3a). A significant impact was observed through one-way ANOVA for FIP uptake (*p* = 0.041). At the same time, a nonsignificant effect was verified for ATZ uptake (*p* = 0.051).

**Figure 3:**
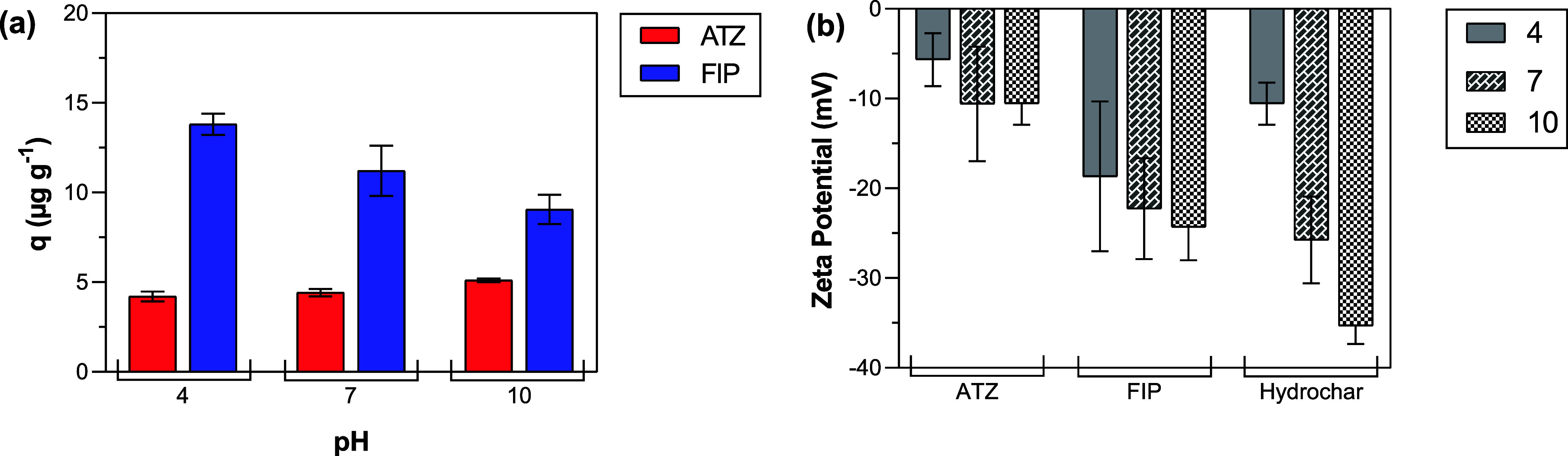
(a) Atrazine (ATZ) e Fipronil (FIP) adsorption by hydrochar at different pH values (4, 7, and 10). The tests were carried out using a pesticide concentration of 100 μg L^–1^, hydrochar mass of 20 mg, agitation of 40 rpm, and contact time of 5 h. (b) Zeta potential measurements of ATZ, FIP, and hydrochar.

ATZ *q*_*t*_ values varied from 4.2 ± 0.3 μg g^–1^ at pH 4 to 5.1 ± 0.1 μg g^–1^ at pH 10. FIP *q*_*t*_ values varied from 13.8 ± 0.6 μg g^–1^ at pH 4 to 9.1 ± 0.8 μg g^–1^ at pH 10. Multiple comparisons were performed using the Tukey test (*p* = 0.05). No significant differences were observed between the pH levels for ATZ (*p* > 0.05). Significant differences in *q*_*t*_ values were observed only for pH 7 and 10 (*p* = 0.035) for FIP.

The ZP values indicate the potential interactions between particles in the solution during the adsorption. Hydrochar exhibited a negative charge in the pH range evaluated (Figure 3b). ZP values varied from −10.6 ± 2.3 mV at pH 4 to −35.3 ± 2.0 mV at pH 10. The hydrochar charge varied from approximately neutral (−10 mV) to strongly anionic (>-30 mV).^37^ The isoelectric point (ZP = 0) of the hydrochar is in an acidic condition (pH < 4), which is the reason that functional groups on the surface were deprotonated (i.e., negatively charged) in the pH range studied. These values fall within the range previously reported for hydrochar from sugar cane bagasse. Zhou et al. (2022)^27^ observed that ZP values of hydrochar varied from +2.4 mV at pH 2 to −34.1 mV at pH 11, with the isoelectric point at pH 2.2.

Both pesticide solutions were negatively charged throughout the evaluated pH range. No significant differences in ZP were observed for ATZ (*p* = 0.333) or FIP (*p* = 0.560). For ATZ, ZP values decreased from −5.7 ± 2.9 at pH 4 to −10.6 ± 2.3 mV at pH 10, whereas for FIP, ZP values decreased from −18.7 ± 8.4 at pH 4 to −24.3 ± 3.7 mV at pH 10. These values are consistent with those reported for different solutions containing ATZ (−15 to −35 mV).^38^ No ZP values were found for FIP solutions in the literature.

The decrease in adsorption efficiency with increasing pH (Figure 3a) may be attributed to the electrostatic repulsive force between the pesticides and hydrochar. Both pesticide and hydrochar charges were more negative at pH 10 than at pH 4 (Figure 3b). Consequently, the negatively charged molecules in the solution experience more repulsion from the hydrochar surface at pH 10, thereby limiting their ability to attach to the surface. The charge impact on adsorbent and pollutant interaction has been previously elucidated.^39,40^

The hydrochar had a better adsorption affinity for FIP than ATZ. FIP is highly hydrophobic (log K_ow_ = 4) with low water solubility (3.78 mg L^–1^ at 20 °C), indicating a stronger tendency to adsorb onto solid surfaces rather than remaining dissolved in water. In contrast, ATZ is moderately hydrophobic (log K_ow_ = 2.61) and has higher water solubility (35 mg L^– 1^ at 20 °C), which contributes to its lower adsorption onto the hydrochar.^41,42^ Sugar cane bagasse has been reported to be hydrophobic in its natural form,^43^ and hydrothermal carbonization can enhance the hydrophobicity of the resulting hydrochar.^44^ Therefore, FIP may be adsorbed by the hydrochar through hydrophobic interactions.

Considering these results, pH 10 was selected to assess the adsorption process under more challenging conditions, such as stronger electrostatic repulsion.

### 3.3 Kinetic Models

Contact time significantly affected the adsorption of both pesticides by the hydrochar (ANOVA test, *p* < 0.0001 for ATZ and *p* = 0.0001 for FIP). The *q*_*t*_ increased from 2.5 ± 0.2 μg g^–1^ (at 0.5 h) to 6.1 ± 0.5 μg g^–1^ (at 48 h) for ATZ and from 5.0 ± 0.4 μg g^–1^ (at 0.083 h) to 11.1 ± 1.8 μg g^–1^ (at 1 h) for FIP (Figure 4). The adsorption equilibrium was reached at 48 h for ATZ and 1 h for FIP, and any further contact time did not increase the adsorption significantly. The time difference to reach the equilibrium for both pesticides may be associated with a stronger tendency of FIP to adsorb onto solid surfaces rather than ATZ, as previously discussed in Section 3.3. Stronger binding affinities typically result in faster adsorption, while weaker affinities allow for more, extending the time needed to reach a stable state.^33^

**Figure 4:**
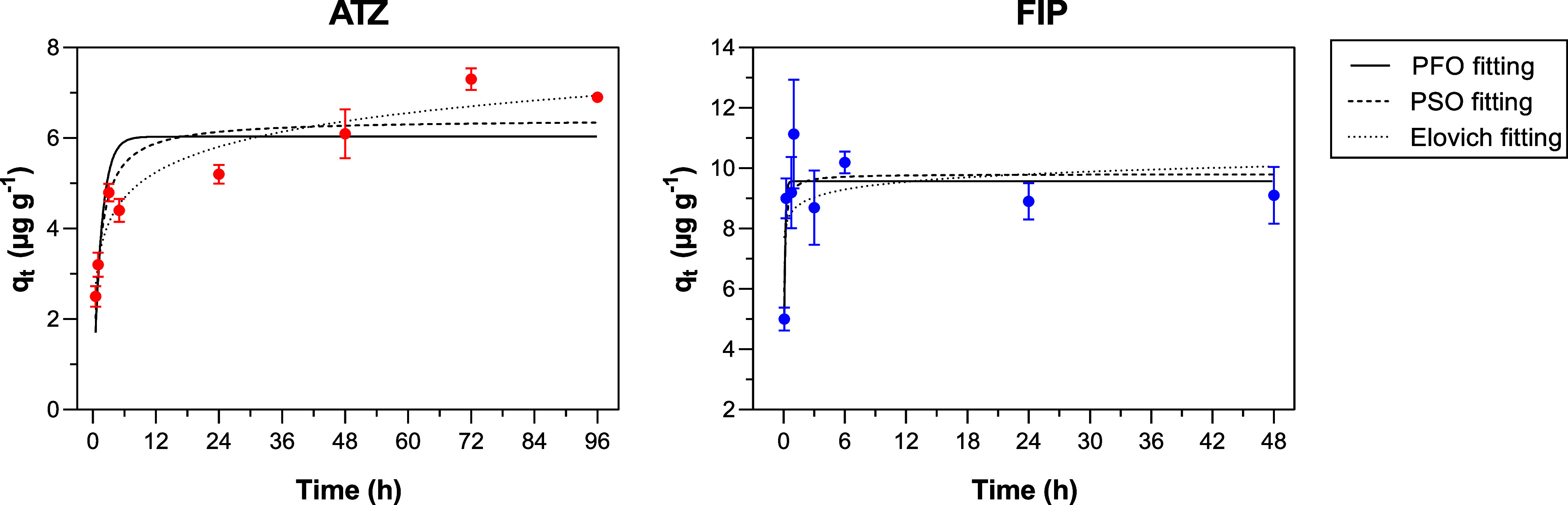
Adsorption kinetics of atrazine (ATZ) and fipronil (FIP) by hydrochar. The tests were carried out using a pesticide concentration of 100 μg L^–1^, a pH of 10, a hydrochar mass of 20 mg, agitation at 40 rpm, and varying contact times (0.5 to 96 h for ATZ and 0.083 to 48 h for FIP).

Experimental data were fitted to PFO, PSO, and Elovich models (Table 2). The goodness of fit was evaluated using the coefficient of determination (*R* ^2^), the chi-square test (χ^2^), and the root mean squared error (*RMSE*). The best fit to the mathematical model is characterized by a high *R* ^2^, combined with low values of χ^2^ and *RMSE.* ^35^

**Table 2:** Values of *q*_*e*_ (μg g^–1^), *K*_*1*_ (h^–1^), *K*_*2*_ (G μg^–1^ h^–1^), *a* (μg g^–1^ h^–1^), and *B* (G μg^–1^), and Values of *R* ^2^, *χ* ^2^, and RMSE for PFO, PSO, and Elovich Kinetic Models.

Pesticide	Model	Model constants	*R* ^2^	χ^2^	RMSE
ATZ	PFO	*q*_*e*_= 6.024, *K*_1_ = 0.651	0.731	0.981	0.858
	PSO	*q*_*e*_= 6.402, *K*_2_ = 0.142	0.848	0.521	0.625
	Elovich	*a* = 41.250, *b* = 1.226	0.937	0.208	0.395
FIP	PFO	*q*_*e*_= 9.541, *K*_1_ = 9.560	0.791	0.753	0.752
	PSO	*q*_*e*_= 9.793, *K*_2_ = 1.896	0.662	1.227	0.959
	Elovich	*a* = 3442.32, *b* = 1.000	0.201	6.199	1.968

The best-fitting models were the Elovich model for ATZ (*R* ^2^ = 0.937) and the PFO model for FIP (*R* ^2^ = 0.791). For ATZ, the Elovich constants *a*, *b*, and *n* were 7.113 μg g^–1^•h^–1^, 7.113 g μg^–1^, and 1.226, respectively. Elovich is an empirical model that describes the kinetics of adsorption processes on heterogeneous adsorbent surfaces. However, it lacks a definite physical meaning.^33^ Thus, it cannot offer important information about the mass transfer mechanisms. For FIP, the PFO constants *q*_*e*_ and *K*_1_ were 9.54 μg g^–1^ and 9.56 h^–1^, respectively. PFO is an empirical model in which the adsorption rate is proportional to the number of unoccupied adsorption sites. It implies that a few FIP molecules can interact with the active sites available in the hydrochar.^33^ The difference in reaching the adsorption equilibrium for both pesticides can also be analyzed by comparing *K*_1_ values from the PFO model. This model’s constant describes how fast the adsorption equilibrium is achieved for FIP compared to ATZ; for example, *K*_1_ values were 0.651 and 9.560 for ATZ and FIP, respectively.

### 3.4 Isotherm Model

ATZ and FIP isotherms are shown in Figure 5. Both isotherms are classified as Type I (convex upward), as they show a tendency toward a horizontal plateau.^34^ Experimental data were fitted to Langmuir, Freundlich, and Sips models (Table 3). The three models fit both pesticides well (*R* ^2^ > 0.93).

**Figure 5:**
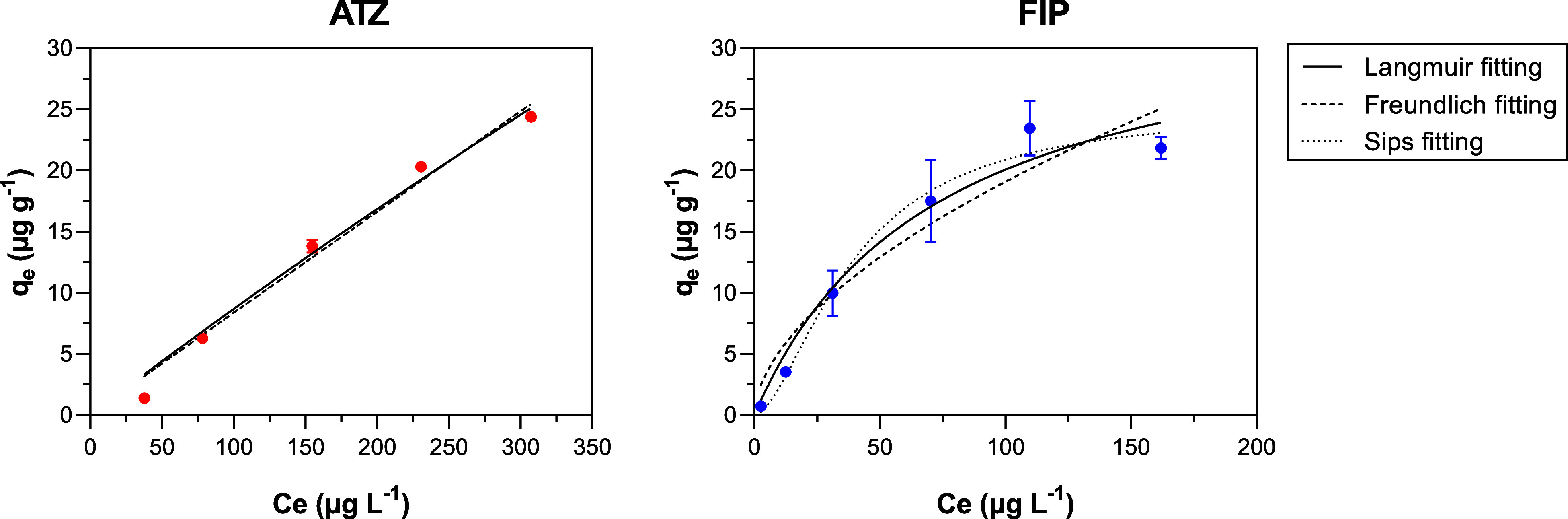
Isotherms of atrazine (ATZ) and fipronil (FIP) adsorption by hydrochar. The tests were carried out using hydrochar mass of 20 mg, pH 10, agitation of 40 rpm, defined contact time (48 h for ATZ and 1 h for FIP), and different pesticide concentrations (40 to 400 μg•L^–1^ for ATZ/FIP).

All models can describe ATZ data, as demonstrated by the high *R* ^2^ value (*R* ^2^ = 0.988) obtained for the three equations. Sips had the best fit for FIP (*R* ^2^ = 0.986). Langmuir represents monolayer adsorption on homogeneous surfaces, while Freundlich characterizes multilayer adsorption on heterogeneous surfaces.^34^ On the other hand, Sips is a hybrid model for heterogeneous surfaces that combines both models.^45^ ATZ adsorption may occur in different forms on the hydrochar surface sites with varying adsorption affinities (Table 3).

**Table 3:** Values of a_m_ (μg g^–1^), K_L_ (L g^–1^), K_F_ [(μg g^–1^) (L μg^–1^)^1/n^], N (−), β_S_ (−), K_S_ (L μg^–1^), and a_S_ (L μg^–1^) and Values of R^2^, χ^2^, and RMSE for Langmuir, Freundlich, and Sips Isotherm Models.

Pesticide	Isotherm model	Model constants	*R* ^2^	χ^2^	*RMSE*
ATZ	Langmuir	a_m_ = 273.284; *K*_*L*_ = 0.0003	0.988	1.592	1.009
	Freundlich	*K*_*F*_ = 0.132; *n* = 1.092	0.988	1.941	1.138
	Sips	*K*_*S*_ = 0.095, β_*S*_ = 0.983, *a*_*S*_ = 0.0002	0.988	1.032	2.129
FIP	Langmuir	a_m_ = 34.63; *K*_*L*_ = 0.0138	0.971	3.573	1.543
	Freundlich	*K*_*F*_ = 1.416; *n* = 1.771	0.931	8.428	2.37
	Sips	*K*_*S*_ = 0.048, β_*S*_ = 1.690, *a*_*S*_ = 0.002	0.986	2.310	1.075

### 3.5 Adsorption Mechanism

Hydrochar and pesticide solutions are negatively charged, indicating that other nonelectrostatic interactions may govern adsorption. The mechanisms often assigned to pesticide adsorption by biochar are hydrophobic interaction, hydrogen bonding, and π–π interactions.^46^

As discussed previously (Section 3.2), pesticides and hydrochar are hydrophobic. ATZ is moderately hydrophobic, while FIP is highly hydrophobic. Thus, hydrophobic interactions are expected to occur during the adsorption. Pesticide molecules can function as both a hydrogen bond donor and acceptor, enabling it to interact with various functional groups through hydrogen bonding.^47^ Additionally, π–π interaction may occur between π-electron-rich adsorbates and π-electron systems on the adsorbent surface.^48^ Atrazine (1,3,5-triazine) and fipronil (phenyl ring) contain aromatic rings in their structures, which create a π-electron system. When the aromatic rings of pesticide molecules approach the aromatic rings on the biochar surface, π-electron clouds interact through π–π stacking.

ATR-FTIR analysis was performed before and after the adsorption of ATZ/FIP to identify the potential functional groups involved in these adsorption mechanisms (Figure 6). A high concentration (1000 μg L^–1^) was employed, in contrast to the concentration used in the adsorption experiments (100 μg L^–1^), to enhance the detection of interactions between ATZ/FIP and the hydrochar surface.

**Figure 6:**
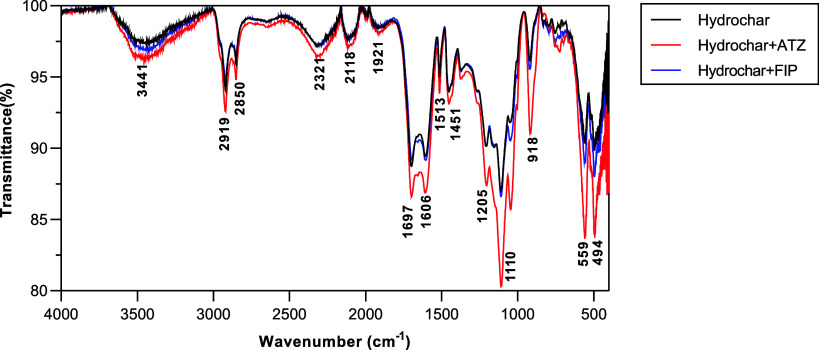
ATR-FTIR spectra before and after the adsorption of ATZ and FIP. The tests were carried out using a pesticide concentration of 1 mg L^–1^, a hydrochar mass of 20 mg, a pH of 10, agitation at 40 rpm, and a contact time of 5 h.

Different functional groups may be involved in pesticide adsorption onto the hydrochar, and bigger peaks were observed after the adsorption. Peak positions generally remained unchanged, and no new peaks were observed after the adsorption.

The peaks of groups with O–H stretching vibrations (3441 cm^–1^) increased, indicating that carboxyl (−COOH) or hydroxyl (−OH) groups were associated with the adsorption. Other bands associated with oxygen functional groups also increased, such as those at 1697 cm^–1^ (C = O), 1110 cm^–1^ (C–O–C), and 1513 cm^–1^ (C–O). It is known that oxygen functional groups (such as hydroxyl and carboxyl groups) on the adsorbent surface can form chemical bonds with the oxygen and nitrogen functional groups in pesticides.^46^ Hydroxyl groups can also form hydrogen bonds between the functional groups in pesticide molecules and the biochar surface when the appropriate geometric and electronic structural conditions are found.^47^

The peaks corresponding to C = C (1606 cm^–1^) and C–O (1513 cm^–1^) shifted after the adsorption. It is assigned to the π–π interaction between the aromatic ring in the pesticide molecule and the π-electron-rich region of the hydrochar (Figure 1).

### 3.6 Literature Comparison

The results reported in the literature for removing atrazine and fipronil by low-cost adsorbents are presented in Table 4. The adsorption capacities reported vary greatly, ranging from 0.158 to 178 mg g^–1^ for ATZ and 0.035 to 0.738 mg g^–1^ for FIP. Direct comparison with the present results is limited because of variations in initial pesticide concentrations and operating parameters. Previous studies used much higher initial concentrations, for example, 0.01–0.4 vs 0.02–2.5 mg L^–1^ for FIP and 0.01–0.4 vs 4–200 mg L^–1^ for ATZ. The range of concentration selected in this study considered a worst-case scenario, i.e., a higher concentration than reported in the literature by 2 orders of magnitude, based on previous studies in real water matrices.^5–9^ The adsorption is driven by the concentration gradient between the pesticide in solution and the adsorbent surface. A higher initial concentration provides a stronger driving force to overcome the mass transfer resistance of pesticides between the aqueous and solid phases, leading to higher adsorption capacities. These findings are supported by Saha et al. (2014),^19^ who reported that the adsorption capacity of fipronil increased from 0.07 to 0.12 mg g^–1^ with an increase in its initial concentration from 2.5 to 10 mg L^–1^.

**Table 4:** Overview of Adsorbents Obtained from Biomass Applied for Pesticide Removal from Aqueous Solutions

Raw material	Activation method	Pesticide	Superficial area (m^2^ g^–1^)	Initial concentration (mg L^–1^); pH	Adsorption capacity (mg g^–1^)	Reference
Rice husk ash	Nitric acid and methanol	Fipronil	-	2.5;-	0.07	^19^
Byproducts of sawmills	Pyrolysis at 350 °C	Atrazine	1.467	4–10; -	0.424	^21^
	Pyrolysis at 450 °C		2.438		0.158	
	Pyrolysis at 550 °C		3.565		0.127	
Corn straw	Phosphoric acid and pyrolysis at 400 °C	Atrazine	573	30; 6.5	26.9	^18^
Araçá fruit husks	Ferric chloride and pyrolysis at 500 °C	Atrazine	431	5–40; pH 7	55.85	^17^
Rice husk	Potassium hydroxide and pyrolysis at 150–200 °C	Atrazine	5.16	30; -	2.18	^20^
Banana Peel Powder		Atrazine	-	20; pH 5.5	3.02	^14^
Açaí pulp	Zinc chloride and pyrolysis at 700 °C	Atrazine	920.56	200; pH 6.5	178	^12^
Sugar cane bagasse fly ash	-	Fipronil	-	0.02; pH 7.2	0.738	^16^
Hackberry seeds	Carbonization and activation with KOH at 300 and 500 °C	Atrazine	3.45	10; pH 2, 4, 6, 7, 8 and 10	107.29	^13^
Sugar cane bagasse and vinasse	Phosphoric acid and hydrothermal carbonization at 230 °C	Atrazine	11.92	0.04–0.4; pH 10	0.273	This study
		Fipronil			0.035	

Further studies are recommended to improve the affinity between the hydrochar and the pesticides studied. Functionalization is often applied to reduce the limitations of adsorbents derived from biomass, such as low pore volume and surface charge.^46^ For instance, this can be achieved by introducing metal ions such as Fe, Ca, Mg, and Ti into the surface and pores of the adsorbent. This modification can improve the catalytic activity, electrostatic attraction, and surface morphology, as previously demonstrated.^49^

## 4 Conclusion

This study confirms the viability of using hydrochar derived from vinasse and sugar cane bagasse as an adsorbent for removing atrazine and fipronil from water. The material exhibited considerable adsorption capacity across a range of pH values. The application of classical kinetic and isotherm models enabled a reliable understanding of the adsorption behavior and mechanisms involved. These findings highlight the potential of valorizing sugar cane industry residues in the development of low-cost treatment strategies for pesticide-contaminated waters. Further optimization of surface properties and selectivity may enhance the performance of hydrochar in future applications.

## Supplementary Material



## Notes

Detailed descriptions of chromatography method validation for fipronil and atrazine quantification (PDF)
