# Supplementary material for: Application of Sugar Cane Bagasse Hydrochar for Fipronil and Atrazine Removal from Water
Source: ACS Omega. 2025 Nov 12;10(46):55544–53. doi: 10.1021/acsomega.5c06438 (PMC12658607; doi:10.1021/acsomega.5c06438)
Supplement: Supplementary file 1 [file ao5c06438_si_001.pdf]

Supplementary Information

Application of sugarcane bagasse hydrochar for fipronil and atrazine removal from water

Avenancia Tavares Belo de Carvalho[a], Luan de Souza Leite[a], Beatriz De Caroli Vizioli[a], Sandro José de Andrade[a,b], Márcia Cristina Bisinoti[c], Cassiana Carolina Montagner[a*]

[a]Environmental Chemistry Laboratory, Institute of Chemistry, University of Campinas, Campinas, São Paulo, 13083-970, Brazil.
[b]Institute of Physics and Chemistry, Federal University of Itajubá, Itajubá, Minas Gerais, 37500-903, Brazil.
[c]Institute of Biosciences, Humanities and Exact Sciences, São Paulo State University (UNESP), São José Do Rio Preto, São Paulo, Brazil.

*Email: ccmonta@unicamp.br

1. METHOD VALIDATION

The analytical method was validated according to the National Institute of Metrology Standardization and Industrial Quality (Inmetro) and the Brazilian Health Regulatory Agency (Anvisa) guidelines.[1,2] The figures of merit evaluated were selectivity, instrumental limit of detection (iLD), instrumental limit of quantification (iLQ), linearity, trueness, precision, and robustness.

1.1.1 Selectivity

The selectivity of the method was assessed by verifying its ability to detect the analytes in the presence of potential interferents (Figure S1). Experiments were conducted using pesticide solutions at pH levels of 4, 7, and 10, both in the presence and absence of hydrochar. For each condition, 20 mg of hydrochar (when applicable) and 5 mL of an aqueous solution at a concentration of 100 µg L^{-1} were used. The samples were agitated at 40 rpm for 5 hours.

Figure S1. HPLC-DAD chromatograms of pesticide solutions at pH 4, 7, and 10 a) in the absence of hydrochar and b) in the presence of hydrochar.

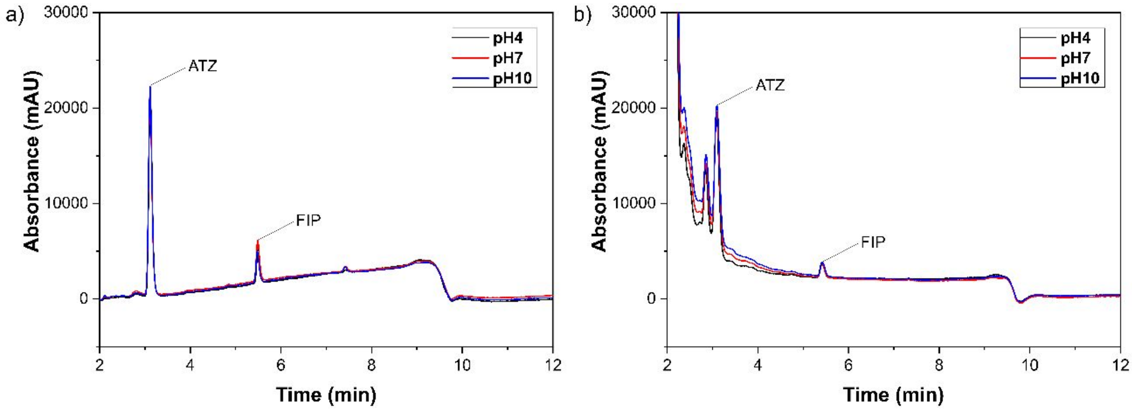

The method demonstrated selectivity, as both ATZ and FIP peaks were observed at their expected retention times in all tested conditions. No interfering peaks were detected in the chromatograms, either in the samples with or without hydrochar, confirming that potential interferents did not affect the identification and quantification of the analytes.

1.1.2 Instrumental limits of detection (iLD) and quantification (iLQ)

The iLD and iLQ were determined through the analysis of standard solutions at progressively lower concentrations of the target pesticides in ultrapure water. The signal-to-noise ratio (SNR) was calculated for each concentration level by comparing the analytical signal to the baseline noise. SNRs of 3:1 and 10:1 were used as the criteria for establishing the iLD and iQL, respectively (Table S1).[1]

Table S1. Instrumental limit of detection (iLD) and instrumental limit of quantification (iLQ).

	**iLD (μg L^{-1})**	**iLQ (μg L^{-1})**
ATZ	2	5
FIP	5	10

1.1.3 Linearity

Linearity was evaluated using eight different concentrations (5, 10, 20, 40, 100, 200, 300, and 400 μg L^{-1}) of standard solutions in ultrapure water, with three replicates for each concentration level. The Grubbs' test (α = 0.05) was used to detect outliers, the Shapiro-Wilk's test (α = 0.05) was used to evaluate normality, the Cochran's test (α = 0.05) was used to evaluate homoscedasticity of data, Analysis of Variance (ANOVA, α = 0.05) was used to evaluate the significance of regression, and the Student's t-test (α = 0.05) was used to evaluate the significance of the coefficients (Table S2).

No outliers were detected by the Grubbs' test for any of the analytes. The Shapiro–Wilk's test indicated that residuals followed a normal distribution for FIP. For ATZ, the residuals did not meet the normality criteria. However, the deviation from normality was slight, and considering the robustness of ANOVA and linear regression to minor violations of normality, no significant impact on the analysis is expected. The Cochran's test confirmed the homoscedasticity of residuals for both analytes, indicating constant variance across calibration levels. ANOVA results demonstrated that the regression models were statistically significant, with both R^2 values exceeding the critical thresholds.[2] The linear coefficients were found to be not statistically significant based on the Student's t-test, supporting the adequacy of the calibration curves.

Table S2. Statistical analysis of linearity.

	ATZ		FIP	
Shapiro-Wilk's test	*W*	*p-value*	*W*	*p-value*
$W_{(0.05;10)} = 0.905$	0.800	0.0003	0.930	0.0967
Cochran's test	*C*	-	*C*	-
$C_{(0.05;8;3)} = 0.653$	0.648	-	0.598	-
ANOVA	R^2	*F*	R^2	*F*
$R^2 > 0.990$; $F_{(0.05;1;22)} = 4.30$	0.9999	206441,42	0.9981	11496,92
Two-tailed Student's t-test (ang. coeff.)	*t*	*p-value*	*t*	*p-value*
$t_{(0.025;22)} = 2.074$	0.420	3.4E-45	107.224	2.1E-31
Two-tailed Student's t-test (lin. coeff.)	**t**	**p-value**	**t**	**p-value**
$t_{(0.025;22)} = 2.074$	454.358	0.6784	1.369	0.1849

1.1.4 Trueness

Trueness was evaluated by analyzing ultrapure water samples spiked at concentration levels of 10, 100, and 400 μg L^{-1}, with three replicates each. The recovery values fall within the acceptable ranges of 60–115% for the 10 μg L^{-1} level and 80–110% for the 100 and 400 μg L^{-1} levels (Table S3).[1]

Table S3. Statistical analysis of method trueness (mean ± standard deviation).

	10 μg L^{-1}	100 μg L^{-1}	400 μg L^{-1}
ATZ	$106._1 \pm 2._3$ %	105.7 ± 0.5 %	104.4 ± 0.5 %
FIP	83.2 ± 3.1 %	$95._5 \pm 2._6$ %	$84._0 \pm 4._0$ %

1.1.5 Precision

Precision was evaluated at three levels: repeatability of injection, repeatability of preparation, and intermediate precision between days.

The repeatability of injection was assessed by performing 10 consecutive injections of the same solution at a concentration of 1 mg L^{-1}. The relative standard deviation (RSD) was 1.0% for ATZ and 3.4% for FIP. These results fall within the acceptable limit of 11% for this concentration level.[1]

The repeatability of preparation was assessed by preparing 10 independent solutions at 100 μg L^{-1} and performing single injections. Intermediate precision was evaluated by repeating the repeatability of the preparation assay on a separate day. For ATZ, the RSD was 0.9% on the first day and 0.8% on the second day. For FIP, the RSD was 1.8% on the first and second days.

The Shapiro-Wilk's test (α = 0.05) was used to assess the normality of data, and the F-test for two-sample variances (α = 0.05) was used to assess the homogeneity of variances between the two groups. Then, the two-tailed two-sample Student's t-test assuming equal variances (α = 0.05) was used to compare the means between the two groups (Table S4). In both cases, the calculated t-values were lower than the critical t-value, indicating that there was no statistically significant difference between the group means.

Table S4. Statistical analysis of method precision.

	ATZ		FIP	
Shapiro-Wilk's test	*W*	*p-value*	*W*	*p-value*
$W_{(0.05;10)} = 0.842$	0.883 (d1)	0.140 (d1)	0.928 (d1)	0.431 (d1)
	0.886 (d2)	0.152 (d2)	0.956 (d2)	0.735 (d2)
F-test	*F*	*p-value*	*F*	*p-value*
$F_{(0.05;9;9)} = 3.18$	1.08	0.4565	1.04	0.4797
Two-tailed Student's t-test (equal var.)	*t*	*p-value*	*t*	*p-value*
$t_{(0.025;18)} = 2.101$	0.201	0.8433	2.030	0.0574

d1: day 1; d2: day 2.

1.1.6 Robustness

The robustness assay was conducted by evaluating the effect of acetonitrile brand (Sigma Aldrich, J.T. Baker, and Carlo Erba), the initial composition of acetonitrile in the mobile phase (58.8%, 60.0%, and 61.2%), and the mobile phase flow rate (0.98, 1.00, and 1.02 mL min^{-1}). The assays were performed using the same standard solution at a concentration of 100 μg L^{-1}.

A one-way ANOVA (α = 0.05) was performed to determine whether these factors caused significant differences in the analytical response (Table S5). Calculated F-values greater than the critical F-value were considered statistically significant.

Among the three factors evaluated, only the mobile phase flow rate showed a statistically significant effect for both pesticides. Thus, a post-hoc Tukey's HSD test ($\alpha = 0.05$) was performed to identify which specific flow rates differed from each other. This multiple comparison procedure allowed a more detailed evaluation of the pairwise differences, confirming which conditions had a measurable impact on the analytical signal. Tukey's HSD test indicated significant differences between all tested flow rates for ATZ. For FIP, significant differences were observed between 0.98 and 1.00 mL min^{-1} and between 0.98 and 1.02 mL min^{-1}.

Table S5. Statistical analysis of method robustness.

	ATZ		FIP	
Factor ($F_{(0.05;2;6)} = 5.14$)	*F*	*p-value*	*F*	*p-value*
Acetonitrile brand	1.86	0.2347	0.12	0.8872
Initial composition of acetonitrile	1.54	0.2894	4.44	0.0655
Mobile phase flow rate	338.62	6.8E-07	17.20	0.0033
Tukey's HSD test for mobile phase flow rate ($q_{(0.05;3;6)} = 4.34$)	*q*	*p-value*	*q*	*p-value*
0.98 vs. 1.00	19.19	0.00002	0.36	0.9820
0.98 vs. 1.02	36.78	7.2E-7	10.33	0.0050
1.00 vs. 1.02	17.59	0.00004	9.97	0.0060

REFERENCES

(1) INMETRO. *Orientação Sobre Validação de Métodos Analíticos: Documento de Caráter Orientativo (DOQ-CGCRE-008)*; 2020. http://www.inmetro.gov.br/Sidoq/Arquivos/Cgcre/DOQ/DOQ-Cgcre-8_08.pdf.

(2) Anvisa. *RESOLUÇÃO DA DIRETORIA COLEGIADA - RDC Nº 166, DE 24 DE JULHO DE 2017*; 2017. https://bvsms.saude.gov.br/bvs/saudelegis/anvisa/2017/rdc0166_24_07_2017.pdf.
